# Habitat environment is associated with the microbiota of the human terminal airway

**DOI:** 10.3389/fmicb.2026.1887778

**Published:** 2026-07-22

**Authors:** Yaohui Sun, Xiaojian Li, Xiaoping Zheng, Xueqian Sun, Jian Liu, Sheng Zhang, Guihong Zhang, Wei He, Wenwen Huo, Jiebin Zuo

**Affiliations:** 1 Department of Thoracic Surgery and Lung Transplantation, The Fifth Affiliated Hospital of Sun Yat-sen University, Zhuhai, Guangdong, China; 2 Guangdong Provincial Key Laboratory of Biomedical Imaging and Guangdong Provincial Engineering Research Center of Molecular Imaging, The Fifth Affiliated Hospital of Sun Yat-sen University, Zhuhai, Guangdong, China; 3 Department of Urology, The Affiliated Changsha Central Hospital, Hengyang Medical School, University of South China, Changsha, Hunan, China; 4 Medical School, Yan’an University, Yan’an, Shanxi, China; 5 Department of Thoracic Surgery, General Hospital of Ningxia Medical University, Yinchuan, Ningxia, China; 6 Department of Thoracic and Cardiovascular Surgery, The Affiliated Hospital of Zunyi Medical University, Zunyi, China

**Keywords:** habitat environment, lung bullae, respiratory microbiome, terminal airway, ultra-deep metagenomics

## Abstract

While environmental exposures are closely associated with the human microbiome, the microbial landscape of the terminal airways remains largely uncharacterized due to the ethical challenges of tissue sampling. To address this gap, we analyzed surgically resected idiopathic lung bullae (localized developmental anomalies surrounded by otherwise normal tissue) to establish a baseline microbiome atlas. We performed ultra-deep metagenomic sequencing on terminal airway tissues from 60 subjects residing in two climatically distinct Chinese cities: Zhuhai (a subtropical coastal region) and Yinchuan (an arid, high-altitude industrial area on the Qinghai-Tibet Plateau). Our analysis revealed that the high-altitude Yinchuan cohort exhibited significantly higher microbial loads and alpha diversity compared to the coastal Zhuhai cohort. Functionally, the Yinchuan microbiome was enriched in taxa associated with fatty acid beta-oxidation, alongside a markedly higher burden of virulence factors and antibiotic resistance genes. These compositional and functional differences may be associated with regional variation in climate, altitude, and local antibiotic usage patterns, whereas the Zhuhai cohort exhibited greater fungal diversity. Ultimately, this study provides the tissue-resolved microbial atlas of the human terminal respiratory tract and reveals substantial differences in microbial composition and function across distinct habitat environments. Furthermore, these findings suggest a potential association between environmental conditions and variation in resident microbiota, providing a basis for future investigations into how environmental change may influence respiratory microecology and human health.

## 1 Introduction

The human body comprises a complex ecosystem inhabited by a myriad of microbial organisms. Studies have elucidated the profound influence of co-resident microbiota on human health and disease, encompassing fundamental physiological processes such as energy metabolism and developmental pathways, as well as the modulation of pathogenic processes (Aggarwal et al., 2023). Recent investigations underscore the pivotal role of environmental factors in shaping the composition of this co-resident microbiome. These factors encompass various elements, including immediate air quality, indoor bio-aerosol microbiota, rural/urban residency, pet ownership, as well as exposure to specific environmental chemicals such as sulfamethoxazole, and pharmaceutical agents like benzylpenicillin and gentamicin (Beghini et al., 2021; Carroll et al., 2020; Chen et al., 2018; Chen et al., 2021). Notably, a recent study discerned substantial disparities in the vaginal microbiomes of women residing at high altitudes compared to those at sea level (Chun et al., 2021).

The human terminal airway denotes the intricate network of bronchioles and alveoli comprising the terminal airway, serving as a direct interface with the external environment. Extensive investigations have unveiled dynamic alterations in the microbiota residing within the terminal airway under various pathological conditions, including neoplastic growth and infectious diseases (Davis et al., 2018; Dickson et al., 2016). However, our understanding of the microbiota inhabiting the terminal airway in human terminal airway remains limited, primarily due to ethical constraints limiting the availability of healthy tissue samples and the inherent challenges associated with accurately delineating microbial signatures from bronchoalveolar lavage fluids (BALF), inevitably contaminated by upper respiratory tract microbes (Eisenhofer et al., 2019).

Idiopathic lung bulla are generally considered localized developmental abnormalities and are often surrounded by lung tissue that appears histologically normal. However, instances of rupture may necessitate surgical intervention (Gacesa et al., 2022; Geva-Zatorsky et al., 2017). Consequently, tissue adjacent to bullae provides a rare opportunity to investigate terminal airway microbiota *in vivo*.

In this study, we sought to investigate the association between habitat environment and microbial composition in the human terminal airway. Therefore, we obtained adjacent healthy lung tissues from a cohort of 60 individuals received surgical treatment in two geographically distinct cities in China. Concurrently, we collected comprehensive environmental and meteorological data from these divergent locales and conducted ultra-deep metagenomic shotgun sequencing to elucidate potential associations.

## 2 Materials and methods

### 2.1 Subjects

The diagnosis of idiopathic pulmonary bulla relies upon internationally recognized criteria (Gacesa et al., 2022). Inclusion criteria encompass individuals who have resided in the study regions for a minimum of 1 year prior to the surgical intervention, of Chinese Han ethnicity, students or non-industrial indoor workers, non-smokers, devoid of antibiotic, steroid, or probiotic administration in the 3 months preceding sampling, and lacking underlying diseases. Pulmonary bullae were located in the subpleural region and were resected together with the surrounding lung parenchyma during surgery. For microbiome analysis, macroscopically normal lung tissue located at least 2 cm from the visible margin of the bulla was sampled from the resected specimen. These tissues contained distal airway and alveolar structures, including terminal bronchioles, respiratory bronchioles, and alveolar ducts, and were therefore considered representative of the terminal airway region (Supplementary Figure 1). Histopathological assessment of excised lung tissue revealed normal findings, and all medical evaluations indicated healthy conditions, except for the presence of the lung bulla. Tissue specimens were procured from Zhuhai, Guangdong Province (ZH), and Yinchuan, Ningxia Province (NX), P. R. China, spanning from January 1, 2023 to June 28, 2023. Informed consent was meticulously obtained from all participants and documented (minors have guardian consent). Post-surgical excision, lung tissue samples were expeditiously stored at -80°C within 20 min to preserve sample integrity before subsequent metagenomic sequencing analysis. Ethical clearance for this study was obtained from the Ethics Committees of the Fifth Affiliated Hospital of Sun Yat-sen University (approval number: No. K119-1, 2022) and the Ethics Committees of the General Hospital of Ningxia Medical University (approval number: No. KYLL-2022-1379) prior to study commencement. The study adhered strictly to the principles outlined in the Declaration of Helsinki.

### 2.2 General information and the environment data of the studied habitat

The map was created using a schematic outline of China to indicate the geographical locations of ZH and YC. Meteorological data for both locations were obtained from the weather website,^1^ while data pertaining to outdoor air pollutants such as SO_2_ and PM2.5 were sourced from https://data.cma.cn/. Additional climatic parameters including annual average low-to-high temperatures, average relative humidity, annual average precipitation, average altitude, atmospheric pressure, longitude, and latitude were gathered from Wikipedia.org.

### 2.3 Metagenomic sequencing and bioinformatics analysis

Metagenomic shotgun sequencing was performed on the Illumina NovaSeq6000 platform (40 Gbp per sample) with paired-end reads of 150 bp (PE150). Briefly, Genomic DNA samples were fragmented to 350 bp size via sonication, followed by end-polishing, A-tailing, and ligation with Illumina sequencing adapters. Index codes were incorporated, and PCR amplification was performed. Library quality was evaluated using the Agilent 5400 system and quantified by quantitative PCR (qPCR) at 1.5 nM concentration.

We utilized Fastp (Kim et al., 2019) for quality control of the raw data. Subsequently, to exclude human-derived sequences, the HISAT2 (Kuhn and Stappenbeck, 2013) software was employed. Following this preprocessing step, Kraken2, leveraging a comprehensive microbial database, was utilized for species identification. The resulting output from Kraken2 (Leung and Lee, 2016) was subjected to classification through Bracken2 (Li et al., 2024), facilitating Bayesian re-estimation of abundance to delineate species composition and relative abundance accurately. To assess potential contamination, six DNA extraction blanks were included as negative controls and processed alongside tissue samples throughout DNA extraction, library preparation, and sequencing. Contaminant taxa were identified using the prevalence-based method implemented in Decontam with a threshold of 0.1. Taxa classified as contaminants were removed before downstream analyses (Li et al., 2015). Functional gene profiling and pathway annotation were performed utilizing HUMAnN 3.0 (Li et al., 2020), aligning sequences with UniRef90 IDs and functional databases. Microbial genome assembly was achieved through MEGAHIT (Lloyd-Price et al., 2016), while MetaGeneMark (Lu et al., 2017) facilitated gene prediction and subsequent translation into amino acid sequences for protein identification. These protein sequences were then aligned against the VFDB_setB database using BLASTp to identify virulence factors. Furthermore, antimicrobial resistance gene identification was conducted using RGI, targeting the Comprehensive Antibiotic Resistance Database (CARD).

### 2.4 Statistical analysis

Microbial alpha and beta diversity analysis for the mycobiome were conducted utilizing the R package Vegan, with permutation-based multivariate analysis of variance (PERMANOVA) applied to assess beta diversity. Clinical data were subjected to statistical analysis using SPSS version 26.0, with count data expressed as frequencies and percentages (%). Categorical variables were assessed utilizing either the chi-squared test or Fisher’s exact test. Non-normally distributed continuous variables between groups were compared via two-sided Wilcoxon rank-sum tests. Correlations between variables were computed using Spearman correlation analysis and visualized using Cytoscape (v3.9.1). To mitigate false positives, *p*-values were adjusted using the Benjamini–Hochberg false discovery rate (FDR). The visualization of results was conducted using R software version 4.2.1 for Mac.

## 3 Results

### 3.1 Participants

A total of 66 participants were initially enrolled in the study. However, samples from 6 individuals did not meet the quality control criteria during metagenomic sequencing and were consequently excluded from further analysis. This resulted in a final cohort comprising 60 qualified samples, with 30 samples each from ZH and YC, respectively. The participant characteristics are outlined in Table 1. Statistical analysis revealed no significant differences between the two groups.

**TABLE 1:** Study subjects.

Clinical features	Total (*n* = 60)	ZH (*n* = 30)	YC (*n* = 30)	*p*-value
Age(years)	28.6 ± 12.3	26.1 ± 9.1	31.2 ± 14.5	0.370
15–25	24 (40.0%)	12(40.0%)	12(40.0%)	1.000
26–60	36 (60.0%)	18(60.0%)	18(60.0%)	
Sex				0.347
Male	47(78.3%)	25(83.3%)	22(73.3%)	
Female	13(21.7%)	5(16.7%)	8(26.7%)	
BMI	21.1 ± 2.8	21.2 ± 2.3	21.0 ± 3.3	0.701
Ethnicity		Han	Han	–
Location of the bulla				0.781 –
Left upper lobe	41(68.3 %)	20(66.7%)	21(70.0%)	
Right upper lobe	19(31.7%)	10(33.3%)	9(30.0%)	
Peri-bulla pathology		Normal	Normal	

BMI, body mass index.

### 3.2 Habitat environment data

ZH is regarded as one of the premier “most habitable” cities in China. Positioned along the coastline of the South China Sea, it boasts a subtropical climate characterized by elevated humidity levels and frequent monsoonal activity. In stark contrast, YC is situated upon the Qinghai-Tibet plateau, characterized by arid atmospheric conditions and a cold climate regime. Historically, YC served as one of China’s heavy industrial bases, and now there are sectors such as energy, materials, and semiconductor industries. Despite their divergent climatic and industrial characteristics, both cities harbor comparable population sizes. Notably, the direct geographical distance separating the two regions exceeds 2000 km (Supplementary Figure 2). Environmental parameters detailing the distinct characteristics of each locale are summarized in Table 2.

**TABLE 2:** Environmental characteristics of ZH and YC.

Environmental factors	ZH	YC	*p*-value
Average annual low temperature (°C)	20.3	3.1	*p* < 0.001
Annual average high temperature (°C)	26.0	15.8	*p* < 0.001
PM2.5 (μg/m^3^)	18.7	33.3	*p* < 0.001
SO_2_ (μg/m^3^)	7.1	14.4	*p* < 0.001
Annual average air quality index	37.8	108	*p* < 0.001
Average altitude (m)	22	1340	*p* < 0.001
Atmospheric pressure (kPa)	1	0.87	*p* < 0.001
Average relative humidity (%)	80	57	*p* < 0.001
Average annual precipitation (mm)	1999.3	186.3	*p* < 0.001
Longitude and latitude	22°16′16″N 113°34′37″E	38°29′15″N 106°13′51″E	–
Population	2,439,585	2,859, 074	–

### 3.3 Diversity analysis

Cumulatively, a substantial dataset comprising 2481.0 gigabase pairs (Gbp) was generated from these 60 samples, with an average sequencing depth of 41.4 Gbp per sample (Supplementary Figure 3A). Saturation analysis conducted to assess sample dilution indicated that both sample size and sequencing depth adequately met the experimental requirements (Supplementary Figures 3B,C). Upon the removal of host genome sequences, each sample yielded an average of 1,514,734 reads (Supplementary Table 1). Overall, the human terminal airway microbiome is predominantly composed of bacteria and fungi, with low levels of archaea and viruses. Notably, the YC region exhibits a higher total microbial load in the adjacent healthy lung tissues compared to the ZH region (Supplementary Figure 3D).

**FIGURE 1:**
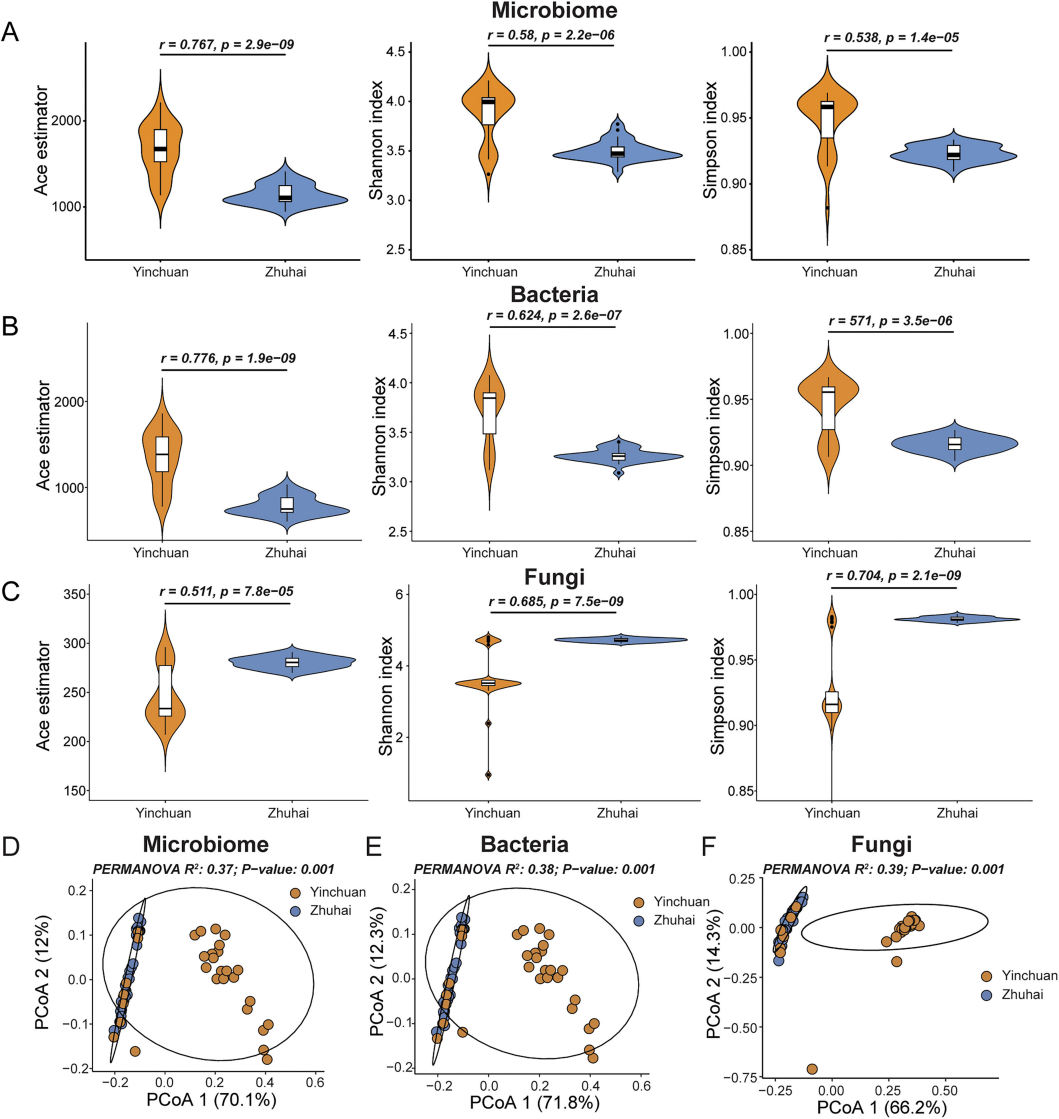
Diversity of the microbes in the adjacent healthy lung tissue samples from ZH and YC. **(A–C)** ACE, Shannon, and Simpson indices for the overall microbiome **(A)**, bacterial communities **(B)**, and fungal communities **(C)** in ZH and YC respectively (Wilcoxon rank-sum test). **(D–F)** PCoA analysis based on Bray-Curtis distances of the overall microbiome **(D)**, bacterial communities **(E)**, and fungal communities **(F)** in ZH and YC.

A comprehensive taxonomic analysis identified a total of 4766 taxa distributed across all samples, spanning 51 phyla, 100 classes, 219 orders, 436 families, 1120 genera, and 2840 species. The suite of diversity metrics employed in our analysis unveiled distinct microbial diversity profiles between the ZH and YC cities. Specifically, the YC city exhibited higher diversity (Shannon index), greater richness (ACE index), and enhanced evenness (Simpson index) compared to ZH (Figure 1A). Notably, analysis focusing on the predominant bacterial component yielded similar findings (Figure 1B). Furthermore, samples originating from the ZH region displayed narrower inter-individual differences compared to those from the YC region. The alpha diversity of the fungal component in the ZH region was significantly higher than that in the YC region (Figure 1C). Subsequent Bray-Curtis distance-based principal coordinate analysis (PCoA) elucidated significant structural disparities not only at the overall microbial community level (Figure 1D) but also within the bacterial (Figure 1E) and fungal (Figure 1F) taxonomic levels. These findings demonstrate the pronounced ecological divergence between the two regions, reflecting distinct microbial community compositions and structural arrangements shaped by local environmental factors and habitat characteristics.

To assess the potential influence of demographic and sampling-related variables, we performed a multivariable PERMANOVA analysis including study region, age, sex, BMI, and tissue sampling location. After adjustment for these covariates, study region remained significantly associated with microbial community composition (*R*^2^ = 0.318, *P* = 0.001). Sex showed a smaller but significant association with community variation (*R*^2^ = 0.035, *P* = 0.040), whereas age, BMI, and tissue sampling location were not significantly associated with microbial composition (Supplementary Table 2).

### 3.4 Compositional analysis

**FIGURE 2:**
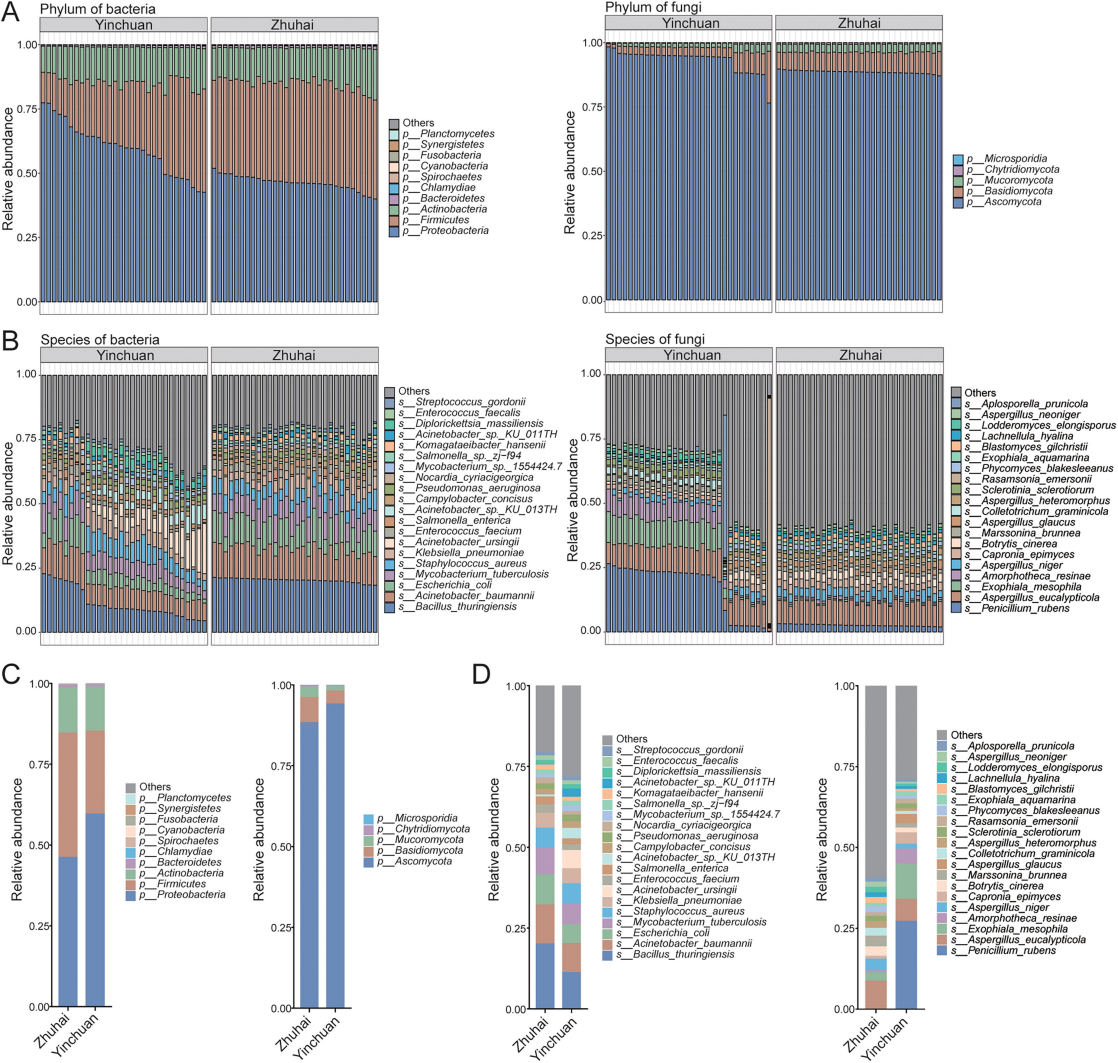
Composition of bacteriome and mycobiome in the adjacent healthy lung tissues at different taxonomic levels in ZH and YC. **(A,B)** represents the composition at the level of phylum **(A)** and species **(B)** for individual samples. **(C,D)** illustrates the average distribution at phylum **(C)**, and species levels **(D)**.

The predominant microbes in the two groups were *Proteobacteria*, *Firmicutes*, and *Actinobacteria*, with *Proteobacteria* comprising approximately half of the bacteriome. The mycobiome is primarily composed of *Ascomycota* (Figures 2A,C). In the bacteriome, the most prevalent genera in both ZH and YC regions are Bacillus, Acinetobacter, and *Mycobacterium* (Supplementary Figure 4A). At the species level, *Bacillus thuringiensis*, *Acinetobacter baumannii*, and *Escherichia coli* were the most prevalent bacteriome species, accounting for 24.7 and 40.0% in ZH and YC regions, respectively (Figures 2B,D). In the mycobiome, *Aspergillus* is predominant in ZH, whereas *Aspergillus*, *Penicillium*, and *Exophiala* are enriched in the YC region (Supplementary Figure 4B). Consequently, significant differences exist between the mycobiomes of the two regions, with *Aspergillus eucalypticola* being the predominant species in Zhuhai, while *Penicillium rubens* predominates in YC (Figures 2B,D). This substantial disparity may be associated with air humidity. Additionally, we observed that the species composition in the population of ZH is more stable, with less inter-individual variation. These findings suggest that while the YC region exhibits greater microbial species diversity, the ZH region harbors a higher proportion of high-abundance species.

**FIGURE 3:**
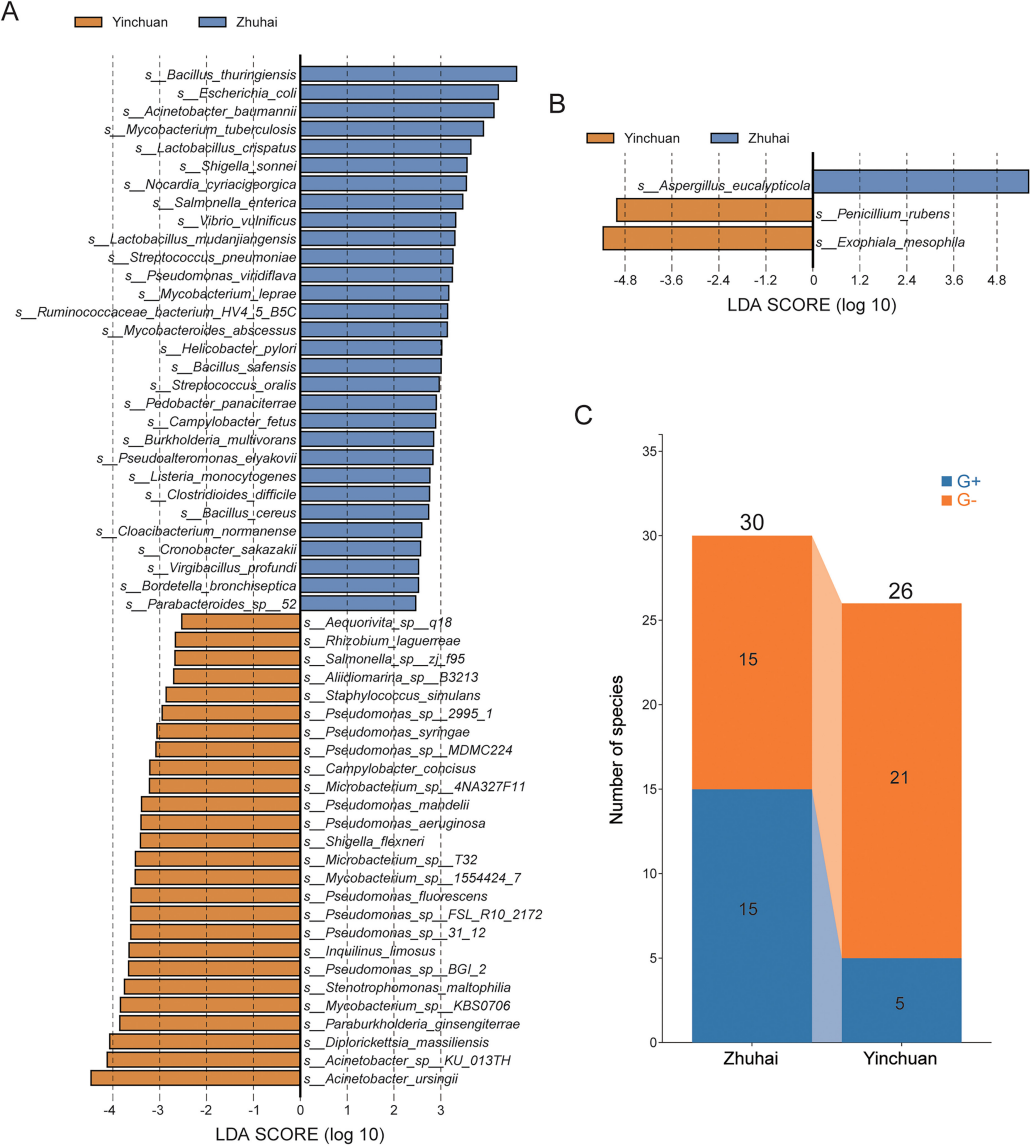
Identification of differentially enriched microbial taxa in ZH and YC through LEfSe. **(A,B)** Differential analysis of bacterial **(A)** and fungal **(B)** taxa between ZH and YC, with blue indicating microbial taxa enriched in ZH and yellow indicating those enriched in YC (LDA score > 2, *P* < 0.05); **(C)** Boxplots representing the distribution of Gram- positive and Gram-negative bacteria in the terminal airway of ZH and YC.

To evaluate the uneven distribution of high-abundance species in the adjacent healthy lung tissues between YC and ZH regions, 547 species with relative abundances greater than 0.1% were selected for differential abundance analysis. Using Linear Discriminant Analysis Effect Size (LEfSe) (Luengo and Olivera, 2020), we identified 30 bacteria and 1 fungus significantly enriched in the ZH region, as well as 26 bacteria and 2 fungi significantly enriched in the YC region (LDA > 2, *p* < 0.05) (Figures 3A,B and Supplementary Table 3). Noteworthy among these enriched species were *B. thuringiensis*, *A. baumannii*, and *E. coli*, which displayed relatively higher abundance in the ZH region, whereas the YC region exhibited enrichment of species such as *Acinetobacter* (Figure 3A). In addition, nine species belonging to the genus *Pseudomonas* were enriched in the YC group. The genus *Pseudomonas* is recognized for its diverse metabolic repertoire, allowing it to thrive across various ecological niches. Furthermore, we also observed a higher abundance of Gram-negative bacteria in the YC region (Figure 3C). Together, these findings indicate distinct microbial compositions between the two regions and are consistent with an association between habitat environment and variation in terminal airway microbiota.

### 3.5 Association between environmental factors and terminal airway microbiota

**FIGURE 4:**
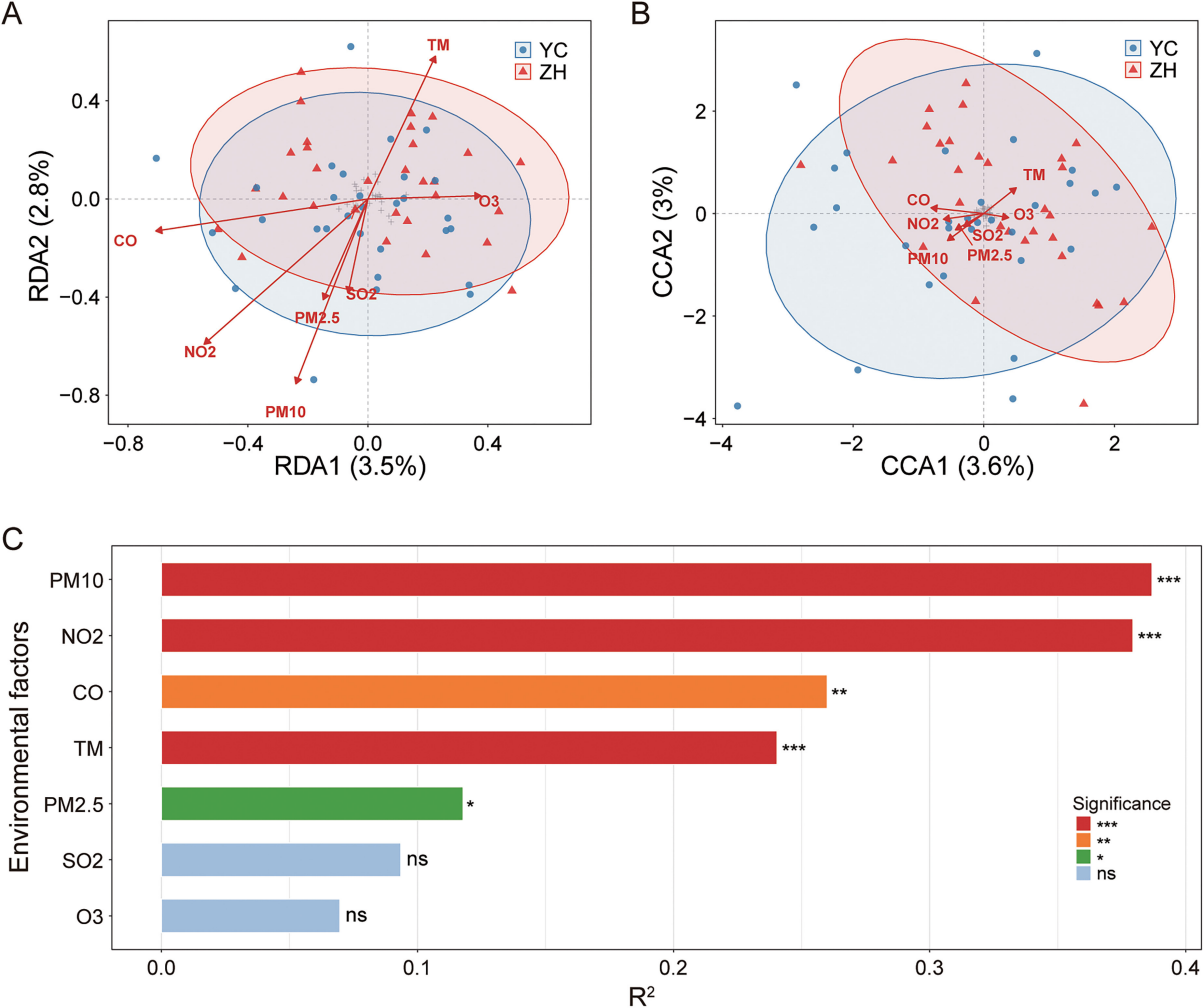
Association between environmental factors and terminal airway microbiota. **(A)** Redundancy analysis (RDA) of microbial community composition and environmental variables. **(A)** RDA of microbial community composition and environmental variables. **(B)** CCA of microbial community composition and environmental variables. **(C)** Explanatory power of individual environmental variables. Permutation tests determined statistical significance, ns, no significant, *P < 0.05; **P < 0.01; ***P < 0.001.

To further examine the relationship between environmental exposure and terminal airway microbiota, redundancy analysis (RDA) and canonical correspondence analysis (CCA) were performed using annual average environmental and meteorological variables, including temperature (TM), PM2.5, PM10, NO_2_, SO_2_, CO, and O_3_. Both analyses revealed clear associations between microbial community composition and environmental factors, with samples from ZH and YC showing distinct distributions along the environmental gradients (Figures 4A,B). Among the variables examined, PM10 and NO2 showed the strongest associations with microbial community variation (*R*^2^ = 0.39 and 0.38, respectively; *P* < 0.001), followed by CO (*R*^2^ = 0.26, *P* < 0.01) and temperature (*R*^2^ = 0.24, *P* < 0.001) (Figure 4C). PM2.5 was also significantly associated with community structure, although its explanatory power was lower (*R*^2^ = 0.12, *P* < 0.05), whereas SO_2_ and O_3_ were not significantly associated with microbial variation. These findings indicate that differences in air quality and climatic conditions were associated with variation in terminal airway microbial communities across the two study regions.

### 3.6 Correlations between species in different habitats

**FIGURE 5:**
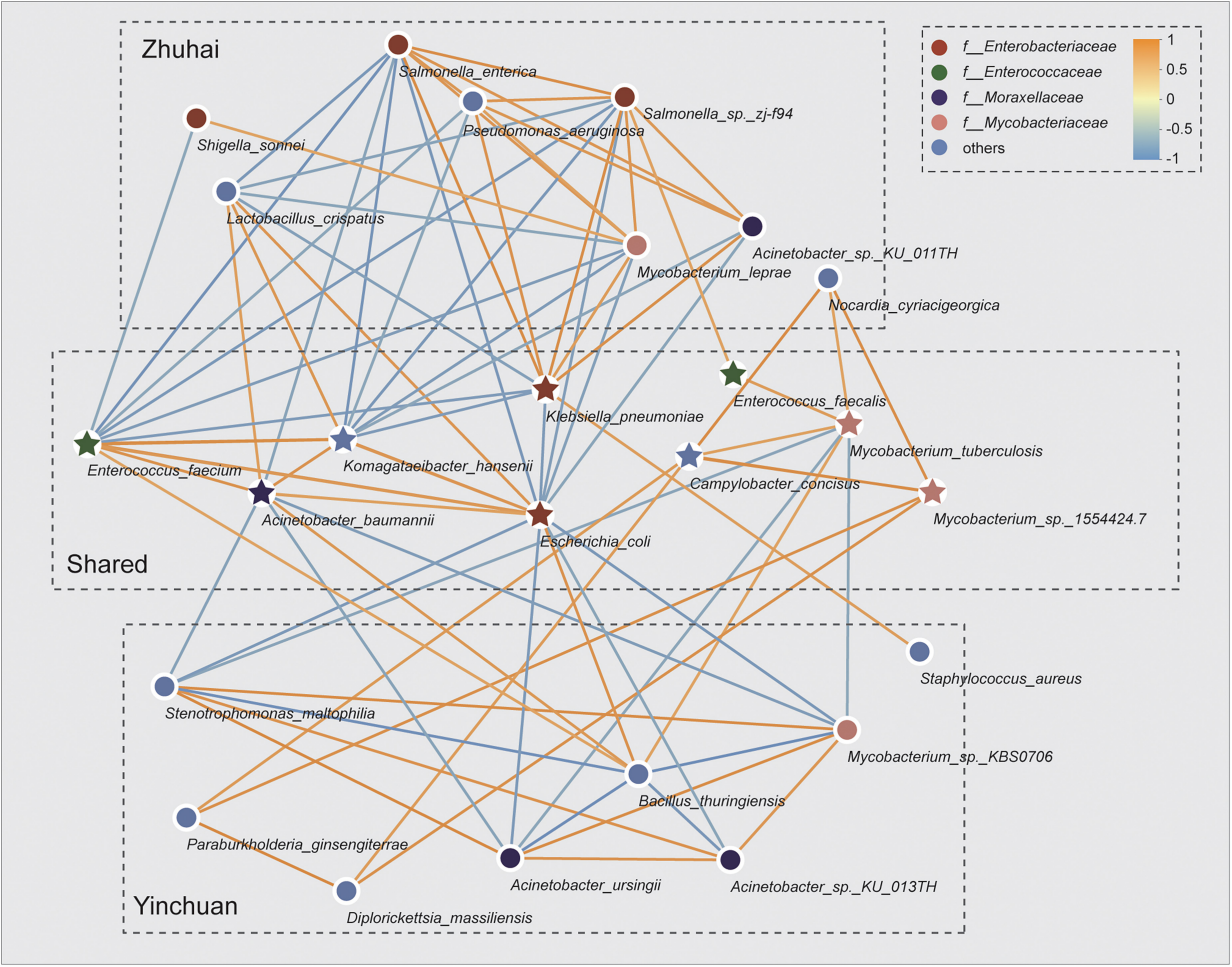
Species correlation networks of terminal airway microbiome between ZH and YC. Nodes of different colors are filled based on family taxonomic levels, correlation coefficients are calculated using Spearman, with yellow indicating positive correlation and blue indicating negative correlation.

We utilized Spearman correlation analysis to construct correlation networks for species with relative abundances greater than 1% in ZH and YC regions, respectively. After retaining correlated pairs with coefficients > 0.7 or < −0.7, we identified 36 (YC) and 52 (ZH) strongly correlated species pairs (Supplementary Table 4). The construction of correlation networks for these associated pairs is depicted in Figure 5. Overall, ZH exhibited more species-strongly associated pairs compared to the YC region. Species such as *E. coli-E. faecium*, *E. coli-A. baumannii*, *E. faecium-K. hansenii*, and *E. coli-K. hansenii* showed similar correlation patterns between the two regions, indicating ubiquitous microbial interactions across different regions. Conversely, species such as *B. thuringiensis*, *K. pneumoniae*, *Acinetobacter ursingii* showed strong correlations with multiple species in the YC region, which were absent or exhibited opposite correlations in the ZH region. Similarly, species such as *P. aeruginosa*, *S. enterica*, *K. pneumoniae*, *K. hansenii*, and *Mycobacterium leprae* displayed similar characteristics in the ZH region, suggesting that microbial interactions are influenced by regional factors. These results indicate that the interaction patterns of terminal airway microbiota in ZH and YC regions are not entirely identical, and geographical location, climate, and environmental factors may influence the structure and interactions of microbial communities in different regions.

### 3.7 Functional analysis

**FIGURE 6:**
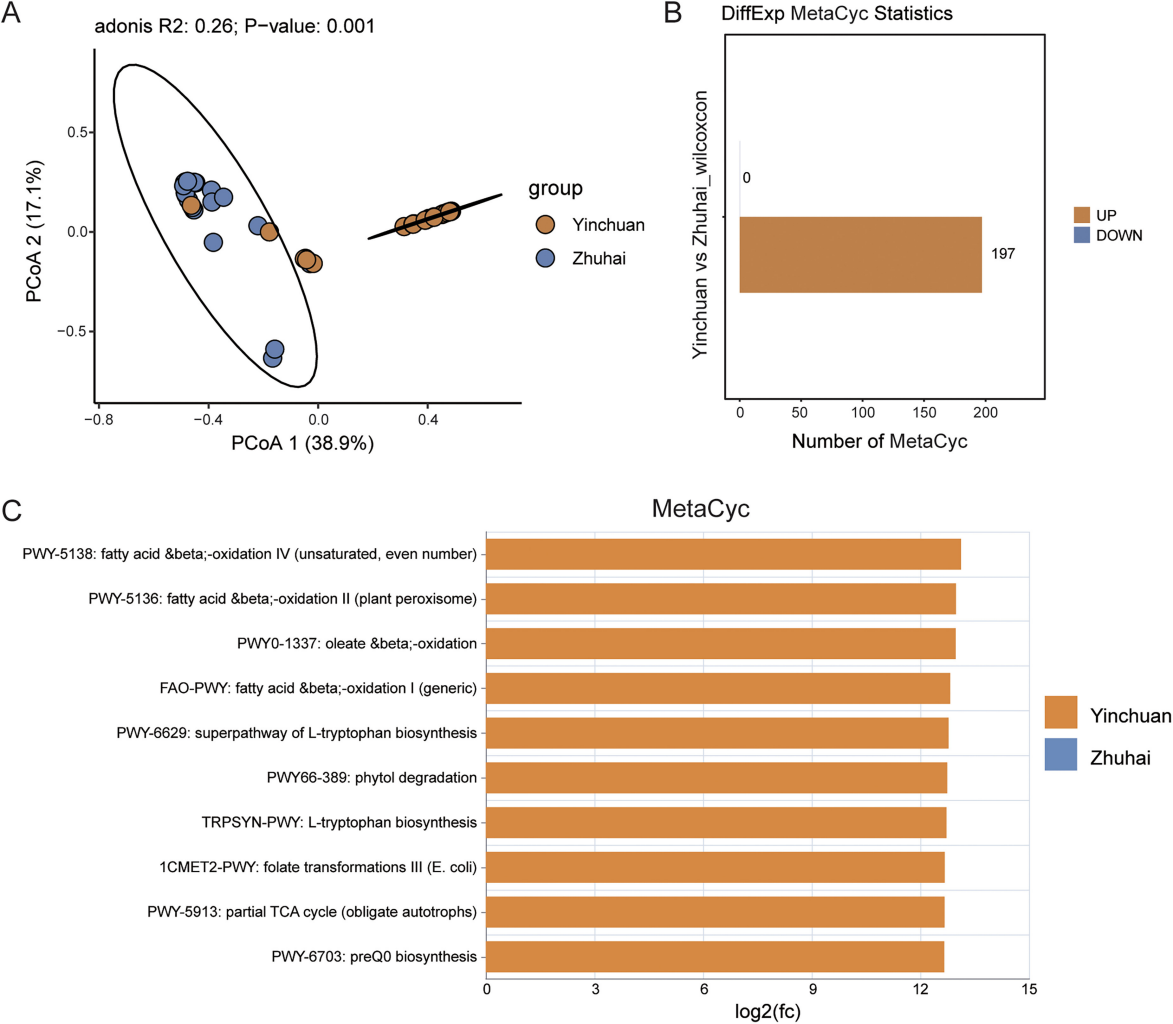
Features of the metabolic functions of the microbes in the adjacent healthy lung tissues in ZH and YC. **(A)** PCoA analysis of MetaCyc metabolic pathways based on Bray-Curtis distances. **(B)** Bar graph representing the numbers of differentially enriched MetaCyc metabolic pathways. **(C)** Positive and negative bar plots representing the top 10 enriched MetaCyc metabolic pathways in YC.

Different habitat environments may lead to metabolic changes in the microbiome of adjacent healthy lung tissue. Therefore, we further analyzed the metagenomic data of individuals from ZH and YC regions from a functional perspective. Annotation of microbial genes to the MetaCyc database identified a total of 197 metabolic pathways. PCoA results revealed significant differences in MetaCyc pathways between ZH and YC regions (Figure 6A). The metabolic pathway differences showed significant enrichment in all metabolic activities in the YC region, especially in fatty acid beta-oxidation (Figures 6B,C). These findings suggest that the functional potential of terminal airway microbiota differs between the two regions and may be associated with environmental variation between the study sites.

### 3.8 Virulence factors and antibiotic resistance genes

**FIGURE 7:**
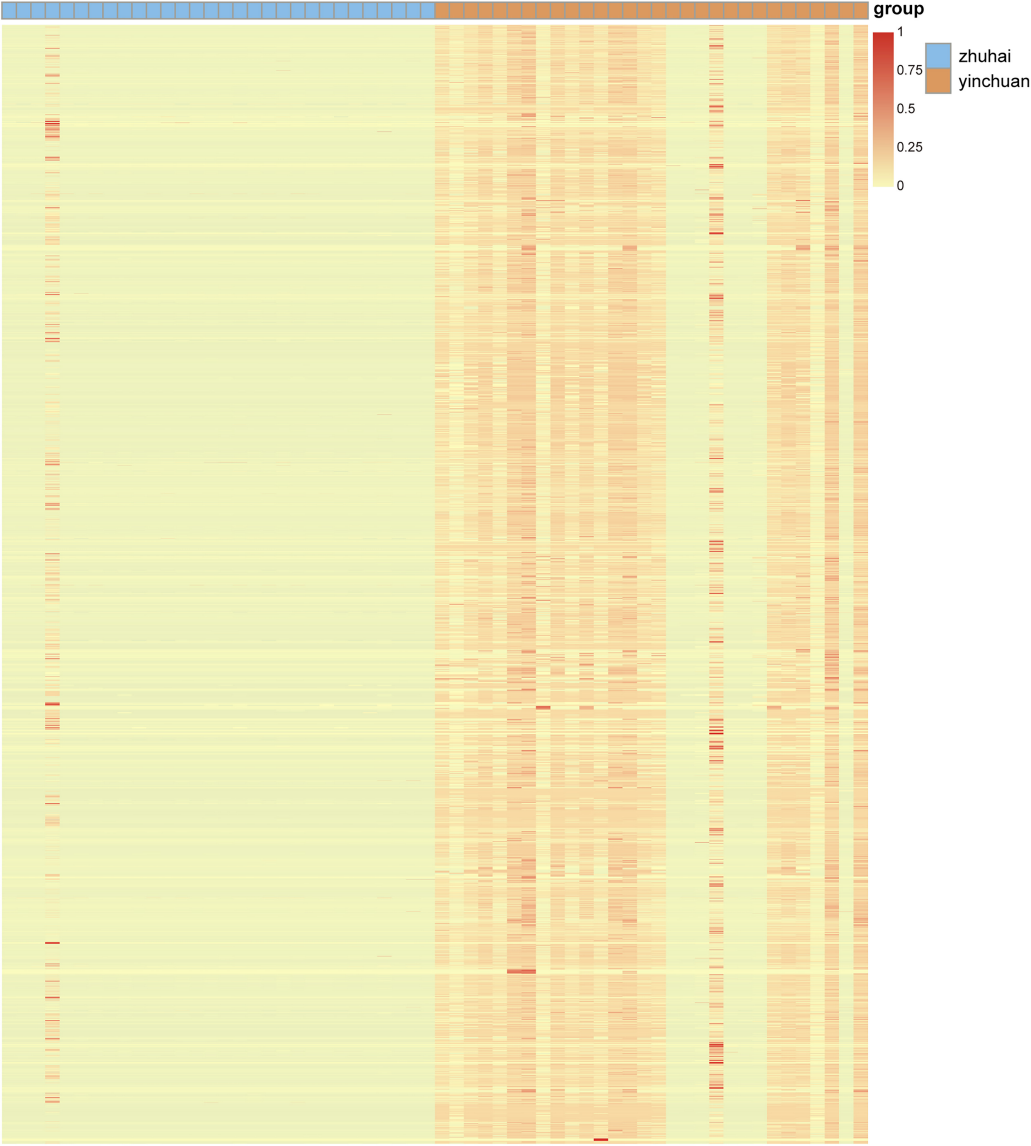
VFs produced by the microbes in the adjacent healthy lung tissues in ZH and YC.

A total of 22,746 genes encoding virulence factors (VFs) were discerned within the dataset. The distribution of these VFs in the samples is shown in Figure 7, with VFs predominantly concentrated in samples from the YC region, suggesting that the harsh habitat may harbor more pathogenic factors producing microbes.

**FIGURE 8:**
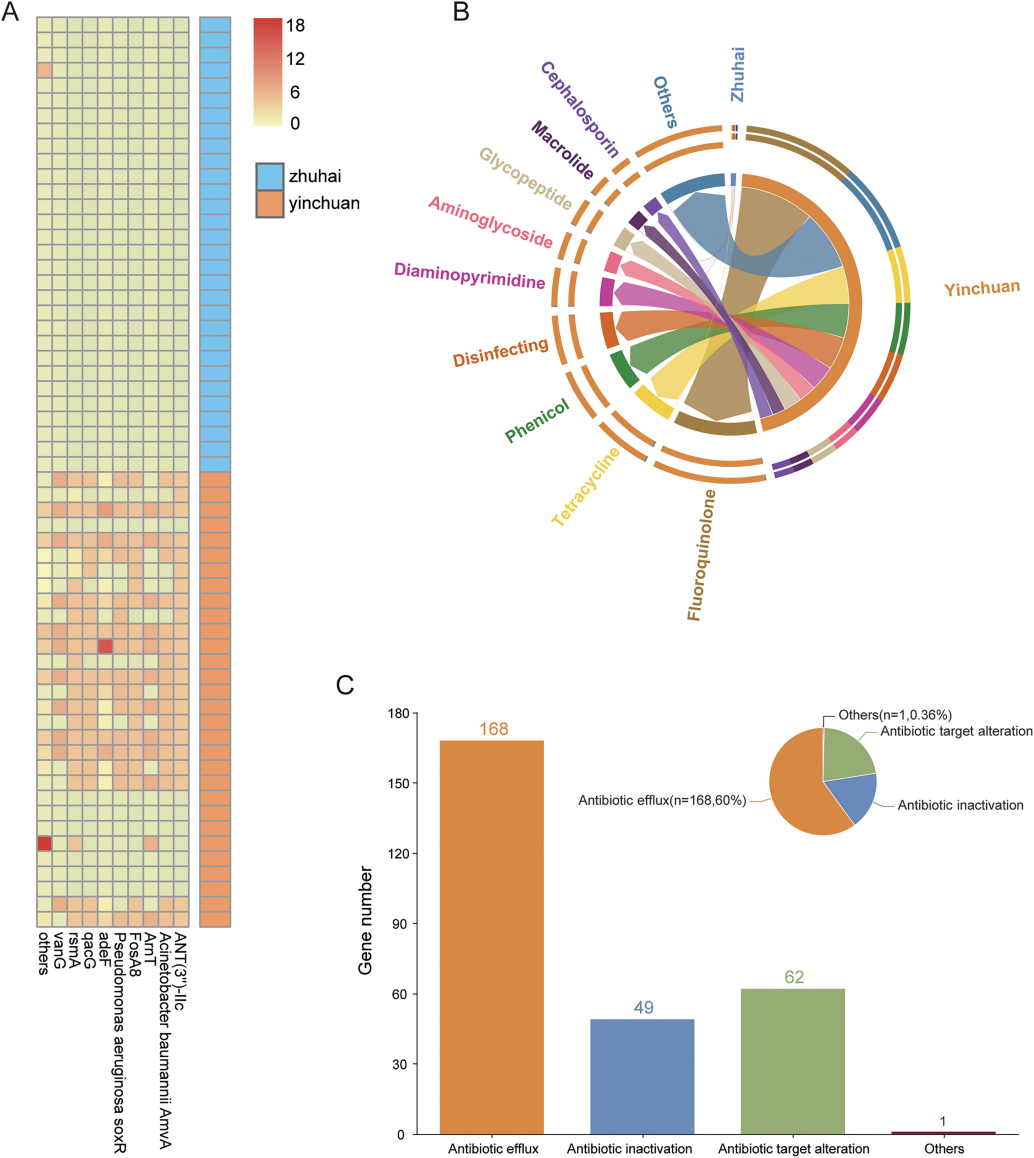
Analysis of ARGs in the respiratory microbiota of ZH and YC. **(A)** Heatmap showing the distribution of AROs in the ZH and YC. **(B)** Circos plot illustrating the types of ARGs. **(C)** Bar and pie charts displaying the predominant mechanisms of action of ARGs in the samples.

Similarly, the presence of antibiotic resistant ontology (AROs) was conspicuously more pronounced in samples obtained from the YC region, whereas only one individual in ZH had enriched AROs (Figure 8A). Regarding the categorization of resistance gene variants, the predominant constituents comprised fluoroquinolone (constituting 22.8% of the total abundance), followed by tetracycline (11.7%) and phenicol (10.4%) variants (Figure 8B). Crucially, the prevalence of these antibiotic resistance genes (ARGs) was predominantly observed among individuals from the YC region. An investigation into the underlying mechanisms precipitating the substantial enrichment of ARGs within the YC region unveiled antibiotic efflux, antibiotic inactivation, and antibiotic target alteration as principal mechanisms, with antibiotic efflux emerging as the most prevalent mechanism (Figure 8C). These findings indicate substantial regional differences in ARG profiles, which may be associated with differences in environmental conditions and local antibiotic exposure between the two regions.

## 4 Discussion

We conducted metagenomic sequencing and association studies utilizing 60 adjacent healthy lung tissue samples sourced from two markedly distinct environmental habitats in China. Our findings present the first microbiome map of near-normal human terminal airways, using histologically normal tissues adjacent to bullae. Our investigation revealed associations between habitat environment and microbial abundance, diversity, and composition, as well as differences in VF and ARG profiles. Notably, this study pioneers the observation that environments characterized by harsh conditions, exemplified by YC, harbor a richer and more diverse microbial ecosystem within the human terminal airways compared to more hospitable locales like ZH. Previous studies have suggested that greater microbial diversity is often associated with increased ecological stability and broader functional potential within microbial communities (Mazumder et al., 2023; Ratajczak et al., 2019). Consistent with this observation, several *Pseudomonas* species were enriched in YC. Given the metabolic versatility of this genus, their increased abundance may contribute to the functional differences identified in the metagenomic analysis, including pathways related to fatty acid β-oxidation (Sahn and Heffner, 2000). In addition, previous studies have linked microbial diversity to immune-related processes and host–microbiome interactions, although the biological implications of the differences observed in the present study remain to be determined (Salter et al., 2014; Schneider et al., 2014; Segata et al., 2011; Shu and Huang, 2022). At the same time, the YC cohort exhibited a higher abundance of virulence factor-associated genes and antibiotic resistance genes. These differences may reflect variation in environmental exposures, microbial community composition, or regional patterns of antibiotic use. However, the present study was not designed to determine the specific factors underlying these observations, and further investigations will be required to clarify the mechanisms involved.

It is worth noting that several taxa identified in this study, including *Acinetobacter baumannii*, *Escherichia coli*, *Klebsiella pneumoniae*, *Pseudomonas aeruginosa*, and *Bacillus thuringiensis*, should be interpreted with caution. These organisms have been reported as both environmental microorganisms and as potential contaminants in low-biomass microbiome studies, where contamination remains an inherent methodological concern despite the use of negative controls and decontamination procedures (Sonnert et al., 2024; Sulaiman et al., 2020). At the same time, their detection does not necessarily indicate contamination. Current ecological models propose that the lower respiratory tract is continuously exposed to microorganisms from the environment and upper airways through inhalation and microaspiration, resulting in the transient presence of taxa that may not establish long-term colonization (White et al., 2014). Therefore, the detection of microbial DNA in terminal airway tissues should not be equated with active infection or stable colonization. In addition, species-level assignments derived from metagenomic sequencing may be influenced by limitations of reference databases, particularly for closely related taxa with high genomic similarity (Whiteside et al., 2021). Consequently, the ecological significance of these organisms in the terminal airway remains uncertain and will require further investigation using complementary approaches such as targeted molecular assays, culture-based validation, and longitudinal sampling.

Several mechanisms may contribute to the regional differences observed in this study. Because the respiratory tract is in continuous contact with the external environment, inhaled air provides a major route through which environmental microorganisms enter the airways. Microorganisms can be transported via aerosols, inhalable particulate matter, and other airborne particles, with potential sources including soil, freshwater, marine environments, and other environmental reservoirs (Wood et al., 2019; Zhang et al., 2023). In addition, microbial transmission across different hosts and ecological niches may further contribute to the diversity of microorganisms encountered by humans (Zhao et al., 2024). Consistent with this concept, previous studies have reported associations between environmental exposures, ecological disturbances, and alterations in lung microbiota composition (Zhu et al., 2010). Together, these observations provide a plausible framework linking environmental variation to differences in airway microbial communities. Consistent with this interpretation, RDA and CCA analyses demonstrated significant associations between several environmental variables, particularly PM10, NO_2_, CO, and temperature, and microbial community composition. Although these analyses do not establish causality, they provide further support for a link between regional environmental conditions and variation in terminal airway microbiota.

To our knowledge, this study provides the first tissue-based characterization of terminal airway microbiota from individuals residing in distinct habitat environments. The observed differences in microbial composition, functional potential, virulence factors, and antibiotic resistance profiles suggest that environmental context may be associated with variation in the terminal airway microbiome. Future studies incorporating additional geographic regions, direct environmental sampling, and longitudinal designs will help clarify the factors contributing to these differences and improve our understanding of how environmental change may influence respiratory microbial ecosystems and human health.

## 5 Conclusion

The habitat environment is intricately linked with the microbial communities cohabiting within the human terminal airway. Host adaptation to distinct environments is putatively associated with variations in the resident microbiota, which may reflect a potential ecological alignment between the host, their lifestyle, and the local environment.

## Data Availability

The raw sequence data reported in this paper have been deposited in the Genome Sequence Archive (GSAHuman: HRA007015) in the National Genomics Data Center, China National Center for Bioinformation/Beijing Institute of Genomics, Chinese Academy of Sciences, and are publicly accessible at https://ngdc.cncb.ac.cn/gsa-human.

## Footnotes

1
https://www.tianqi.com/
