# Supplementary material for: Habitat environment is associated with the microbiota of the human terminal airway
Source: Front Microbiol. 2026 Jul 22;17:1887778. doi: 10.3389/fmicb.2026.1887778 (PMC13439231; doi:10.3389/fmicb.2026.1887778)
Supplement: Supplementary file 1 [file Table_2.docx]

**Supplementary figures**

**Figure S1. Anatomical schematic of the resected lung tissue.**

**Figure S2. Geographical location of ZH and YC.**

**Figure S3. Data quality control results of terminal airway samples from ZH and YC.** (A) Filtered reads passing through after quality control for terminal airway samples from ZH and YC; (B) Specaccum species accumulation curves observed in the two sample groups; (C) Sparse curve analysis of observed species count; (D) Overall component distribution observed in the two sample groups.

**Figure S4. Composition of terminal airway microbiome at different taxonomic levels.** (A-B) Represent species composition at the class (A) and order (B) levels, with the left showing the distribution of different taxonomic units per sample and the right bar plots displaying mean values at the taxonomic level.
